# Secret and Proprietary

**Published:** 1898-02

**Authors:** 


### "Secret" and "Proprietary."

There seems to be a great misunderstanding and misuse of these two terms. Some persons use them as if they were interchangeable, and even make the word nostrum synonymous. It would appear unnecessary to say that everything that is advertised must be owned by some one, have somebody as proprietor, i. e., be "proprietary" in order to have some one to pay for the advertisement. Chairs and bicycles and cod-liver oil, if they have the distinctive name of a certain maker attached to them, are proprietary preparations. We have heard copyrighting spoken of as if it were something wrong and shameful, whereas in itself it has no ethical significance whatever. It is only a brand of the manufacturer. It is the possible secrecy of the copyrighted article or the abuse of the method of copyrighting that makes wrong. In reference to drugs, for example, the manufacturers may conceal the nature of the ingredients, and such things then become secrets; in this case we say it is unprofessional to use or to advertise them.—*Philadelphia Medical Journal.*

[**The following** will explain itself. All over Texas similar action is being had.—Ed.]

*To the Honorable Commissioners' Court of Van Zandt County, Texas:*

Gentlemen:—We, the undersigned physicians, residents of this county, who pay our pro rata of tax to support our county and State, believing that the late law taxing the physicians on their occupation is wrong in spirit, and no expedient arising to justify a State or county in taxing its citizenship for the privilege of working for nothing (we believe doctors do more charity practice than they do paying practice).

Therefore we petition your honorable body for redress as far as in your power lies, and ask that you remove the part of this tax imposed by the county. (Signed by the physicians of the county.)

